# The impact of COVID-19 pandemic on the epidemiological characteristics of permanent dental injury in Xi’an of China: A retrospective study

**DOI:** 10.1097/MD.0000000000035358

**Published:** 2023-09-29

**Authors:** Yang Yang, Yanli Liu, Ziheng Wang, Qiang Li, Jiang Wang, Min Zhang

**Affiliations:** a State Key Laboratory of Oral & Maxillofacial Reconstruction and Regeneration & National Clinical Research Center for Oral Diseases & Shaanxi International Joint Research Center for Oral Diseases, Department of General Dentistry & Emergency, School of Stomatology, The Fourth Military Medical University, Xi’an, Shaanxi, China; b School of Computer Science and Engineering, Beihang University, Beijing, China.

**Keywords:** coronavirus disease 2019, dental trauma, epidemiological characteristics, follow-up, permanent tooth

## Abstract

The coronavirus disease 2019 (COVID-19) has become a major global concern, seriously affecting the lives and health of the population. This retrospective study aimed to investigate changes in permanent tooth injury in the Xi’an area of China influenced by the COVID-19 pandemic. The medical records of 466 dental emergency patients in 2019 and 2020 were retrospectively analyzed. The number of injured teeth in a single patient, the number of injury types, the time from injury to hospital visit and follow-up visits within 1 year before and after the pandemic were analyzed using the chi-squared test and the paired *t*-test. There was significant difference in the number of emergency patients and trauma types before and during COVID-19 pandemic (*P* < .05). The time from injury to hospital visit and the interval between the trauma event and visit showed longer during the COVID-19 pandemic (*P* < .05). The rate of on-time follow-up in the following year was significantly decreased, and the rates of delayed visits and patients lost to follow-up were significantly increased (*P* < .05). The outbreak of the COVID-19 pandemic brought a certain change in the epidemiological characteristics of dental injury in the Xi’an area of China. Dental emergency departments should provide even more timely and effective treatments. It is also necessary to strengthen public education, with emphasis on the importance of timely medical treatment and regular follow-up for dental trauma.

## 1. Introduction

The coronavirus disease 2019 (COVID-19) pandemic spread rapidly within a short time at the beginning of 2020 and has become a global public health event, exerting substantial effects on all aspects of human social life.^[1]^ The virus causing COVID-19 has officially been named 2019-nCoV by the World Health Organization.^[2]^ COVID-19 is highly infectious.^[3]^ Therefore, in Announcement No. 1 issued by the National Health Commission of the People’s Republic of China, it is classified as a category B infectious disease, and it is prevented and treated through prevention and control measures used for category A infectious diseases.^[4]^ COVID-19 has become a major global concern at present, substantially impacting the lives and health of the population.

Dental trauma, a common disease encountered in the dental emergency department, is characterized by sudden occurrence, complex types, diverse treatments, and unstable prognosis; therefore, assessments of its diagnosis, treatment, and prognosis are difficult. The prognosis of patients with dental trauma is greatly affected by timely diagnosis and early treatment. For instance, patients with accidental pulp exposure of young permanent teeth are highly less likely to suffer long-term pulp necrosis if they receive direct pulp capping or vital pulpotomy as soon as possible after trauma.^[5]^ In the case of avulsion injuries, affected teeth are more likely to achieve periodontium healing after prompt reimplantation within 30 minutes after trauma.^[6]^ The COVID-19 epidemic inevitably impacted people’s life habits.^[7]^ Routine medical procedures were also changed to some extent due to the needs of epidemic prevention and control.

There are little reports on the effect of COVID-19 on dental trauma and has a lack of comparison with the situation before the COVID-19 pandemic. The impact of COVID-19 on the epidemic characteristics of dental trauma, visiting habits of the patients is still unknown. In this study, the emergency department visits of patients with dental trauma at the Stomatological Hospital of the Fourth Military Medical University within 1 year (February 2020–January 2021) after the initial stage of the COVID-19 pandemic were reviewed and compared with those 1 year before (February 2019–January 2020). This retrospective epidemiological study was designed to evaluate the effect of the pandemic on permanent tooth trauma in the Xi’an area of China.

## 2. Materials and Methods

### 2.1. Data sources

The study was conducted as a retrospective overview study including all patients with a dental trauma of the permanent teeth who presented the emergency department of the Stomatological Hospital of the Fourth Military Medical University, which is an important dental emergency center in northwestern China. The emergency center is open from Monday to Sunday from 0:00 to 24:00 every day. Information, including patient ID, sex, age, visit time, cause of injury, positions of injured teeth, diagnosis, and the time and number of follow-up visits in the following year collected from February 2020 to January 2021 were enrolled through the information-based electronic medical record system of the hospital. In order to better compare the changes of dental emergency patient visits before and during the epidemic, and avoid the influence of time, season and other mixed factors, we set the data during the same period from February 2019 to January 2020. Medical records were retrieved from the electronic patient registry. The data provided was registered by 26 practitioners of varying backgrounds in regard to their specialization during their routine shifts at the Department of Emergency Dental Care. Upon entering the Unit of Dental Emergency, every patient was asked to grant consent to medical treatment and data processing, and, during the COVID pandemic, another consent form for epidemiological COVID data was introduced.

### 2.2. Diagnoses and classification

These patients mainly complained of dental trauma and met the criteria in the 2020 International Association for Dental Traumatology (IADT) guidelines for the diagnosis^[8,9]^ (infraction, uncomplicated crown fracture, complicated crown fracture, uncomplicated crown-root fracture, complicated crown-root fracture, root fracture, alveolar fracture, concussion, subluxation, lateral luxation, intrusive luxation, extrusive luxation, avulsion) and treatment of dental trauma. Patients with incomplete initial medical records or trauma to the primary dentition were excluded.

### 2.3. Statistical analysis

Data was performed on a Microsoft Excel 365 spreadsheet. The statistical analysis was performed with SPSS 17.0 software (SPSS, Chicago, IL), and charts were plotted using GraphPad Prism 8.0 (GraphPad Software, San Diego, CA). The *χ*^2^ test and the paired *t*-test were used to analyze the distribution before and after the pandemic. The test level was set to *α* = 0.05, and *P* < .05 suggested a statistically significant difference.

### 2.4. Ethical approval

This study was approved by the Institutional Review Board (IRB), School of Stomatology, Fourth Military Medical University (approval number:IRB-REV-2020063). The study was performed in accordance with the ethical standards laid down in the 1964 Declaration of Helsinki and its later amendments. The requirement for informed consent was waived owing to the retrospective nature of the study.

## 3. Results

**Figure 1.**
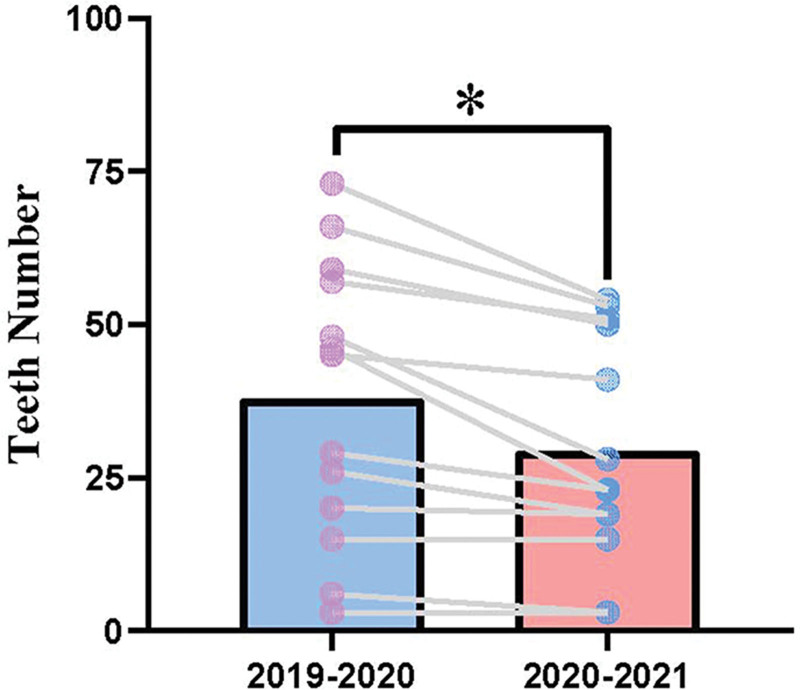
The number of teeth with various types of trauma among patients who visited the emergency department before and during the COVID-19 pandemic. * indicates a significant difference (*P* = .002).

A total of 466 emergency patients and 875 teeth were included in the study, consisting of 257 and 209 patients (493 and 382 teeth) before and after the COVID-19 pandemic, respectively. All trauma types in the international classification of dental trauma were identified in patients with dental trauma who were treated in the Dental Emergency Department of the Stomatological Hospital from February 2020 to January 2021. Damage to tooth tissues accounted for 38.6% of dental trauma patient visits, of which the incidence rates of uncomplicated and complicated crown fractures were highest (13.9% and 13.1%, respectively). Damage to periodontal tissues accounted for 55.5% of injuries, among which extrusive luxation and avulsion injury had the highest incidence rates (14.1% and 13.4%, respectively). Compared with the same period 1 year previously, the number of injured teeth decreased (*P* < .05) (Fig.1). Regarding the proportion of dental trauma types, the proportions of avulsion, uncomplicated crown fracture, and complicated crown fracture increased during the COVID-19 pandemic, while the proportions of infraction and concussion decreased (Table 1).

**Table 1:** Comparison of the number of teeth with different types of dental trauma before and during the COVID-19 pandemic.

Injury type	February 2019 to January 2020		February 2020 to January 2021	
	Teeth N	N (%)	Teeth N	N (%)
Infraction	6	1.1	3	0.8
Uncomplicated crown fracture	66	13.4	53	13.9
Complicated crown fracture	59	12	50	13.1
Uncomplicated crown-root fracture	3	0.6	3	0.8
Complicated crown-root fracture	20	4.1	19	5
Root fracture	26	5.3	19	5
Alveolar fracture	29	5.9	23	5.9
Concussion	48	9.7	28	7.3
Subluxation	45	9.1	41	10.7
Lateral luxation	46	9.3	23	6
Intrusive luxation	15	3.1	15	4
Extrusive luxation	73	14.8	54	14.1
Avulsion	57	11.6	51	13.4

It is shown that the time from injury to hospital visit was longer during the COVID-19 pandemic. The proportion of patients (2.9%) who visited doctor within 0.5 hours after trauma during the COVID-19 pandemic was significantly lower than that (4.7%) 1 year before (*P* < .05), whereas the proportion of patients who visited the emergency department more than 4 hours after trauma (32.1%) was obviously higher than before (26.8%) (*P* < .05, Table 2).

**Table 2:** Comparison of the time from injury to the emergency department visit before and during the COVID-19 pandemic.

Visit time	February 2019 to January 2020 [(n)%]	February 2020 to January 2021 [(n)%]	Change [(n)%]
≤0.5 h	12 (4.7)	6 (2.9)	−6 (−50)
0.5–1 h	41 (16)	35 (16.7)	−6 (−14.6)
1–1.5 h	5 (1.9)	5 (2.4)	0 (0)
1.5–2 h	48 (18.7)	40 (19.1)	−8 (−16.7)
2–2.5 h	1 (0.4)	2 (1)	1 (50)
2.5–3 h	52 (20.2)	32 (15.3)	−20 (−38.5)
3–4 h	29 (11.3)	22 (10.5)	−7 (−24.1)
>4 h	69 (26.8)	67 (32.1)	−2 (−2.9)
Total	257	209	−48

*Notes*: Comparison of the visit times after trauma between the 2 time periods (*P* = .036).

**Figure 2.**
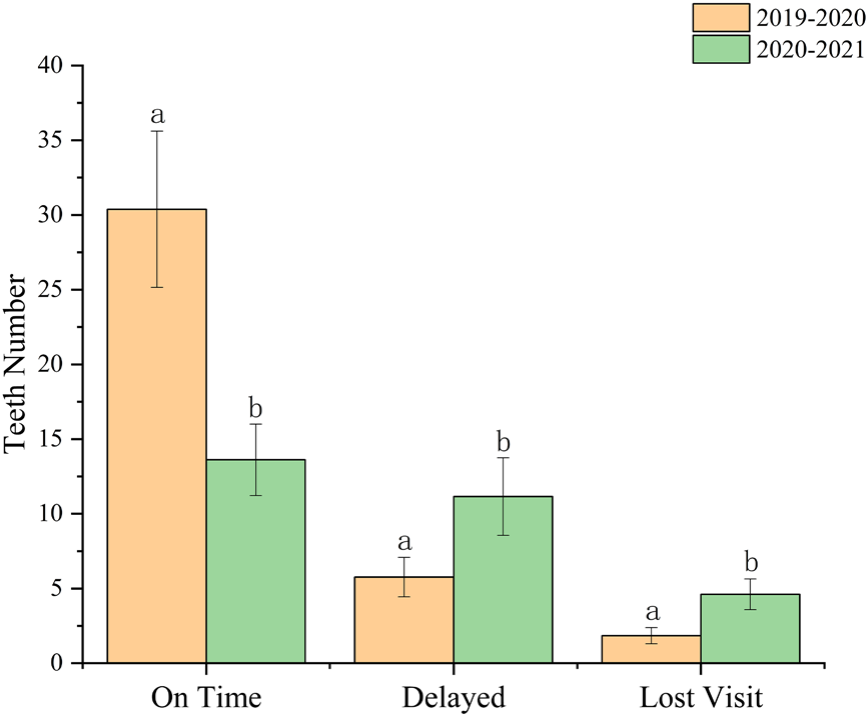
The follow-up visit rates including on-time, delayed and lost visits in the year before and during the first year of the COVID-19 pandemic. Different letters indicate a significant difference (on time: *P* = .000; delayed: *P* = .002; lost visit: *P* = .000).

From February 2019 to January 2020, 80.1% (n = 395) of the 493 affected patients returned to the emergency department for the follow-up visit on time according to the follow-up interval as specified by the IADT. Among the patients who returned on time, the highest return rate was found for those with extrusive luxation (n = 64, 16.2%), followed by avulsion (n = 51, 12.9%), and complicated crown fracture (n = 45, 11.4%). A total of 74 affected teeth belonged to patients with delayed return, accounting for 15%, with the highest rate being for teeth involving uncomplicated crown fracture (n = 19, 25.7%), followed by complicated crown fracture (n = 10, 13.5%), and extrusive luxation (n = 7, 9.5%). In addition, the rate of loss to follow-up was 4.9%, among which patients with uncomplicated crown fracture were the most common (29.2%), followed by complicated crown fracture (16.6%), and concussion (12.5%). From February 2020 to January 2021, the rate of on-time return visits in the following year was obviously decreased compared to the same period 1 year previously (*P* < .05). The rates of delayed visits and patients lost to follow-up were obviously increased and showed significant differences compared to the same period 1 year previously (*P* < .05) (Fig. 2). More precisely, from February 2020 to January 2021, on-time follow-up visits to the emergency department in the following year, according to the IADT visit time, were recorded among patients who accounted for 46.3% (n = 177) of the 382 affected teeth. Among the patients with affected teeth who returned on time, the highest return rate was for injuries involving avulsion (n = 29, 16.4%), followed by extrusive luxation (n = 27, 15.4%), and subluxation (n = 22, 12.4%). Patients with a total of 145 affected teeth exhibited delayed returned, accounting for 38% of injuries, with the highest rate involving extrusive luxation (n = 26, 17.9%), followed by uncomplicated crown fracture (n = 25, 17.2%), and complicated crown fracture (n = 24, 16.6%). In addition, the rate of loss to follow-up was 15.7%, among which uncomplicated crown fracture accounted for the highest proportion of injuries (25%), followed by complicated crown fracture (15%) and concussion and lateral luxation (8.3%) (Table 3).

**Table 3:** Proportions of on-time, delayed and lost follow-up visits within 1 year for different types of dental trauma before and during the COVID-19 pandemic.

Injury type	February 2019 to January 2020			February 2020 to January 2021		
	On time (n%)	Delayed (n%)	No visit (n%)	On time (n%)	Delayed (n%)	No visit (n%)
Infraction	4 (1%)	1 (1.4%)	1 (4.2%)	0 (0)	1 (0.7%)	2 (3.3%)
Uncomplicated crown fracture	40 (10.1%)	19 (25.7%)	7 (29.2%)	13 (7.3%)	25 (17.2%)	15 (25%)
Complicated crown fracture	45 (11.4%)	10 (13.5%)	4 (16.6%)	17 (9.6%)	24 (16.6%)	9 (15%)
Uncomplicated crown-root fracture	2 (0.5%)	1 (1.4%)	0 (0)	1 (0.6%)	0 (0)	2 (3.3%)
Complicated crown-root fracture	15 (3.8%)	3 (4.1%)	2 (8.3%)	10 (5.6%)	5 (3.4%)	4 (6.7%)
Root fracture	20 (5.1%)	5 (6.7%)	1 (4.2%)	12 (6.8%)	3 (2.1%)	4 (6.7%)
Alveolar fracture	23 (5.8%)	5 (6.7%)	1 (4.2%)	14 (7.9%)	6 (4.1%)	3 (5%)
Concussion	39 (9.9%)	6 (8.1%)	3 (12.5%)	13 (7.3%)	10 (6.9%)	5 (8.3%)
Subluxation	39 (9.9%)	5 (6.7%)	1 (4.2%)	22 (12.4%)	15 (10.3%)	4 (6.7%)
Lateral luxation	39 (9.9%)	5 (6.7%)	2 (8.3%)	10 (5.6%)	8 (5.6%)	5 (8.3%)
Intrusive luxation	14 (3.5%)	1 (1.4%)	0 (0)	9 (5.1%)	4 (2.8%)	2 (3.4%)
Extrusive luxation	64 (16.2%)	7 (9.5%)	2 (8.3%)	27 (15.4%)	26 (17.9%)	1 (1.6%)
Avulsion	51 (12.9%)	6 (8.1%)	0 (0)	29 (16.4%)	18 (12.4%)	4 (6.7%)
Total	395 (80.1%)	74 (15%)	24 (4.9%)	177 (46.3%)	145 (38%)	60 (15.7%)

## 4. Discussion

Since the beginning of the COVID-19 pandemic, transmission control measures have been implemented worldwide. This is the largest quarantine attempt in human history to prevent the transmission of an infectious respiratory disease.^[10]^ Dental traumatology is a widespread public healthcare problem. It can be influenced by various factors, such as socioeconomic status or environment. The prognosis of traumatically injured teeth is substantially affected by the visit time after trauma and timely and correct treatment.^[11]^ In the present study, the emergency department visits of patients with dental trauma to the Stomatological Hospital of the Fourth Military Medical University during the COVID-19 pandemic were investigated and analyzed. Relevant data were compared with those from the same period 1 year prior to explore the effect of the COVID-19 pandemic on permanent tooth trauma in Xi’an, China, and to identify both changes in the epidemiological characteristics of dental trauma under the COVID-19 pandemic and possible new challenges to dental trauma treatment.

A certain change in the epidemiological characteristics of dental trauma was confirmed in this study. Compared with the same period before the COVID-19 pandemic, the proportion of avulsion was increased, but the proportions of infraction and concussion were decreased. The possible reasons for these proportions are as follows. First, there are some limitations to this research. It was conducted in a single center and was therefore limited to a subpopulation, and the data evaluated were recorded by different healthcare professionals, leading to possible bias. Second, certain minor dental injuries, such as enamel infraction and concussion, may tend to be overlooked in the diagnosis when other more severe dental trauma is present. Some studies in the literature also support this speculation.^[12,13]^ Third, the number of minor injuries, such as enamel infraction and concussion, was underestimated. Finally, due to the difficulty in seeking medical treatment during the pandemic and because these minor injuries caused only minor discomfort, patients may have chosen personal observation instead of going to the hospital immediately.

The visit time after dental trauma and follow-up visits are important for the prognosis of traumatically injured teeth.^[14–16]^ Timely treatment and follow-up visits after injury can greatly improve the survival rate of injured teeth. The results of this study indicated that most patients visited a doctor within 1 to 3 hours after trauma, but many patients still visited a doctor more than 4 hours after trauma, representing an increase compared with that in the same period 1 year prior. From February 2020 to January 2021, the rate of on-time return visits in the following year clearly decreased, and the rates of delayed visits and patients lost to follow-up clearly increased. These significant differences may be related to the effect of the pandemic. During the pandemic, transportation was not as convenient and fast as that in the past due to travel and traffic control measures used to control the outbreak of COVID-19. Therefore, patient visits were delayed to some extent, especially for patients in surrounding counties and cities, who are often diagnosed and treated after several referrals. Besides, during the follow-up period of 1 year after the end of treatment, the probability of complications was low due to the perfect and accurate treatment of the affected teeth. The traumatized teeth have no obvious uncomfortable symptoms, coupled with the impact of inconvenient intercity-travel during the pandemic for the patients to visit back, the rate of on-time return visits clearly decreased, and the patients lost to follow-up clearly increased.

In addition, we found little change in the effect of the pandemic on the type of dental injury in patients with on-time return visits and lost to follow-up. No matter before and after the COVID-19 pandemic, extrusive luxation and avulsion were the 2 types of injuries with high incidence in those patients who accomplished scheduled follow-up visits; however, the uncomplicated crown fracture, complicated crown fracture and concussion were the 3 main types of injuries in the patients who lost their follow-up. The reason might be that understanding of dental trauma is not comprehensive for the common people, which suggests that the public awareness of dental trauma still needs to be strengthened.

In public opinion, when people can intuitively see the condition of the affected teeth (e.g., gum bleeding symptoms) with injuries such as avulsion and extrusive luxation, patients and their families consider that the trauma is serious; so the compliance of the patient will be better in the entire treatment process and follow-up. However, with injuries such as uncomplicated crown fracture and concussion, due to the mild disease symptoms, relatively simple treatment process and low incidence of subsequent complications, these make the public lack understanding and believe that these types of injuries are not serious, thus greatly affecting their rates of readmission, even in the same period before the COVID-19 pandemic. It also suggested to us that in order to improve the follow-up rate and success rate of dental trauma, we should strengthen the popularization of the knowledge and increase the understanding of dental trauma with the public.

## 5. Conclusions

Since the outbreak of the COVID-19 pandemic, there have been certain changes in the number of patients with dental trauma in Xi’an of China, as well as in the structure and proportion of dental trauma types. More importantly, the COVID-19 pandemic has affected the timely treatment of patients with dental trauma to some extent. We emphasize that attending dentists should provide more timely, effective treatment services for dental trauma patients. It is also necessary to strengthen public education emphasizing the importance of timely medical treatment and follow-up to improve the prognosis of dental trauma.

## Acknowledgments

This study was supported by research funds from the National Clinical Research Center for Diseases Project (LCA202007) and Shanxi Science and Technology Innovation Team Project (2021TD-46).

## Author contributions

**Conceptualization:** Min Zhang.

**Data curation:** Qiang Li, Jiang Wang.

**Formal analysis:** Yang Yang, Yanli Liu, Ziheng Wang, Min Zhang.

**Funding acquisition:** Min Zhang.

**Methodology:** Yang Yang, Yanli Liu, Ziheng Wang, Min Zhang.

**Writing – original draft:** Yang Yang, Yanli Liu, Ziheng Wang.

**Writing – review & editing:** Qiang Li, Jiang Wang, Min Zhang.
